# Acute Pancreatitis

**Published:** 1908-07

**Authors:** Charles G. Stockton, Herbert U. Williams

**Affiliations:** University of Buffalo, Medical Department; University of Buffalo, Medical Department


### BUFFALO MEDICAL JOURNAL.

VOL. LXIII. JULY, 1908. NO. 12

### ORIGINAL COMMUNICATIONS.

### Acute Pancreatitis.

**Report of Four Additional Cases with Autopsies.**

BY CHARLES G. STOCKTON, M. D., AND HERBERT U. WILLIAMS, M. D.
University of Buffalo, Medical Department.

ALTHOUGH the number of cases of this affection that have been reported is now very large, so much about it remains obscure that additional reports still seem desirable. The present writers have already published together accounts of three cases[1]; for this reason those considered here are designated Cases IV. to VII.

CASE IV.—On Friday, February 13, 1903, Dr. Stockton visited at noon in consultation with Dr. Joseph Fowler, Mr.— of Adams, Mass. Mr.— left home Monday morning in his usual health, but when he reached Albany he began to suffer with severe abdominal pain. This became so intense that before he reached Buffalo he was in a condition of great prostration, and it required his utmost power to control himself until his arrival. At Rochester, he wired Dr. Fowler to meet him on his arrival in Buffalo. He was taken from the train in a wheel chair, helped into a carriage, driven to Dr. Fowler's office where morphine was administered, and he reached his stopping place a very ill man. He had a temperature of 102°, with a weak, intermittent pulse. There was vomiting, and intense pain in the median line above the umbilicus, which to some extent radiated to the back. He soon rallied although the pain continued and occasionally the vomiting recurred until Thursday night, when it ceased. Early Friday morning his bowels moved as the result of calomel and salts, they having been confined for forty-eight hours previously. There was moderate tympanites at this time, a great deal of distress, and a sense of great weariness. After the bowels had moved, the pulse became extremely weak and frequent, and jaundice made its appearance; vomiting did not recur.

---

1. American Journal Medical Sciences, 1895, Vol. CX., p. 251; Transactions, Association American Physicians, 1895, Vol. X., p. 227; Philadelphia Medical Journal, 1900, Vol. VI. These three cases were considered together in the Report of the Pathological Laboratory of the University of Buffalo, No. 1.

Dr. Fowler saw the man during the afternoon. He was alarmed by his prostration and Dr. Stockton was asked to see the patient with him. The patient was a very large man, six feet tall, weighing 240 pounds, with much abdominal fat. There was considerable tympanites, tenderness in the median line above the umbilicus, especially on deep pressure. There was very little abdominal tension save that caused by tympany; but the abdomen was so fat that palpation was unsatisfactory. The area of hepatic dullness was slightly increased, the spleen not large. The face and extremities were markedly congested, showing poor oxygenation. The jaundice was most evident over the sclerotics; tongue moderately coated, not characteristic. The patient was lying on his right side, he moved about when requested, but complained of great weakness. He said that in other respects he felt better than in the morning. The pulse could not be precisely counted, although it was above 160; his temperature was 99°. The heart sounds were tapping in quality, first and second sounds alike, nearly inaudible. The respiratory sounds were very feeble, and a few moist rales could be detected at the base of the right lung. A specimen of the urine was asked for, but it could not be obtained. There was a history of what was supposed to be gallstone colic, occurring about nine months before. All things considered, it was believed that the patient suffered from acute hemorrhagic pancreatitis, possibly with gallstone complications. Surgery was advised provided the man's condition sufficiently improved to warrant it. The attending physician had stimulated the patient fully, but no response was seen. These measures were continued, but no improvement resulted and death occurred four and one-half hours later.

His family physician, Dr. A. J. Bond, of Adams, Mass., reports that during March, 1902, the patient was under treatment for indigestion and biliousness, believed to have resulted from sedentary habits and over-indulgence at the table. From April 5th to June 7th, the patient was confined to his bed because of epigastric pain and sensitiveness, with a temperature of 102.5°, the fever remitting in character; there were nausea, vomiting, clay-colored stools, bile-stained urine and anorexia. After a few days the sensitiveness was confined to the region of the gall-bladder. No tumor was discoverable, perhaps owing to obesity of the patient. Subsequent to this the patient was in good health, previous to the illness already recounted.

Autopsy, four hours after death: Rigor mortis and post-mortem lividity well marked; skin slightly jaundiced; a quantity of water, probably not large, had been introduced into the arterial system by the undertaker. The body was that of a large and stout man; subcutaneous fat an inch to an inch and a half thick; abundant subperitoneal and omental fat. The brain and spinal cord were not examined. The contents of the thorax, the spleen, kidneys and urinary bladder were not remarkable. The abdomen was tympanitic. There was no exudate in the general peritoneal cavity and no peritonitis. The stomach and duodenum tore easily. The stomach, duodenum, vermiform appendix and the remainder of the intestines were apparently normal. The liver was large (about five pounds), somewhat soft, slightly mottled. The gall-bladder was very small and contracted, its interior sacculated, and it contained numerous small, soft calculi. The beginning of the cystic duct was obstructed by one of these calculi. The cystic, hepatic and common bile-ducts were all large, open and free from calculi. The main pancreatic duct was of good size, open, and joined the common bile-duct shortly before entering the duodenum. The pancreas was large, hard, and mottled with spots of hemorrhage and fat-necrosis. The head of the pancreas was hard, swollen, and hemorrhagic, and there was a large hemorrhagic infiltration behind it and the duodenum. The distal part of the pancreas appeared small and abnormally fibrous. There were numerous areas of fat-necrosis about the pancreas, especially the head of the pancreas, also in the mesentery and mesocolon, and a few in the omentum, but none in the anterior abdominal adipose tissue. The fat-necrosis was very pronounced at the root of the mesocolon. The lesser peritoneal cavity contained several ounces of slightly blood-stained fluid with floating drops of oil. No obstruction of the large bloodvessels of the pancreas was found.

Cultures made from the lesser peritoneal cavity yielded bacillus coli communis. A diplo- or streptococcus was recovered from the gall-bladder. Examination of sections of the pancreas and its ducts for bacteria was negative, except that in a single field a few small bacilli were found in a thrombus in a vein. Many similar thrombi were examined with negative results. In some situations so much fragmentation of the nuclei of leukocytes had occurred as to render the examination for bacteria very unsatisfactory. The connective tissue of the pancreas was increased in amount, and was abnormally cellular. Small hemorrhages were present in it. Areas of pigmentation were seen, consisting of yellowish-brown granules and fine crystals, largely enclosed in phagocytic cells. In the head of the pancreas there was much exudation of fibrin and the connective tissue contained numerous polynuclear leukocytes; in certain areas there was pronounced fragmentation of the nuclei of the leukocytes. The veins, especially the small veins, frequently contained thrombi, in which leukocytes were prominent. The adipose tissue around the pancreas and between its lobules showed numerous areas of fat-necrosis, frequently surrounded by leukocytes; among the latter fragmentation of nuclei was often conspicuous. The epithelium of the ducts of the pancreas was in good condition, except in the duct of Wirsung where it was wanting, probably owing to manipulation at the autopsy. The epithelium of the acini of the pancreas was of normal appearance in most of the sections, but in some parts areas of considerable size occurred where the nuclei refused to stain. In such parts the nuclei of the ducts also failed to stain. The areas of necrosis were not prominent in the head of the pancreas, but were large in the body. Small and beginning foci of necrosis of the acini were seen sometimes in immediate contact with foci of fat-necrosis. Histological examination of the other organs yielded no important information except in the case of the liver. The hepatic cells showed marked fatty infiltration. The connective tissue contained many leukocytes. Many small bile-ducts were filled with polynuclear leukocytes and granular debris. The impression produced by all the features of this case taken together was that a certain degree of inflammation had been operating in the pancreas for some time upon which the acute fatal process had suddenly supervened. On the whole evidences of acute inflammation were quite marked in this case.

Case V.—In September, 1902, M. W., a stout, well-educated Irish housekeeper, about 50 years old, was seized with intense pain in the epigastric region. She had suffered several hours when Dr. Stockton examined her. There were nausea and some vomiting, temperature of 102°, pulse 120, great depression; the abdomen was moderately distended, not rigid, but sensitive to pressure in the median line just above the umbilicus. There was absence of tenderness in the region of the gall-bladder and the appendix. Dr. Stockton had known the patient for several years, and she had never had a similar attack. She showed more prostration, more abdominal shock than usually occurs in inflammatory processes. Obstruction of the bowels, perforative appendicitis, and perforation or obstruction of other abdominal viscera were considered. The conclusion was that she was probably suffering from acute pancreatitis. She was given a small dose of morphine hypodermically, was relieved and continued to improve. In three or four days, although she showed the effect of her prostration she was free from abdominal symptoms. The nature of the attack was on the whole not clear.

About a year later, in June, 1903, she suffered her final illness which began precisely like the preceding attack. In this last illness she was not under our personal observation. She is stated to have experienced sudden, sharp, severe pain about two o'clock in the morning, with nausea and vomiting. About nine o'clock she was seen by Dr. C. S. Jones, who found the pain so severe that he administered morphine hypodermically; but little relief was felt, and a second hypodermic was given. The pain was located over the stomach, but was not localised so definitely but that both appendicitis and gallstones were considered as possible causes. On the whole the indications seemed to point to intestinal obstruction. Her pulse was feeble, the temperature not notably elevated. Her mind was apprehensive. During the day she was removed by Dr. Jones to the Sisters of Charity Hospital. Her pulse continued to be very rapid, the temperature slightly elevated; there was still vomiting, the face appeared pinched, there had been no movement of the bowels. On the second day of the attack the abdomen was opened by Dr. S. Y. Howell.

No obstruction of the intestines could be found, although the coils of intestine were distended and hyperemic. Areas of fat-necrosis were seen in the omentum. The woman remained in a collapsed condition, and died in about three days.

Autopsy, June 27, 1903; dead 20 hours; the usual rigor mortis and lividity: a linear wound was present in the middle line, beginning one inch above the symphysis and extending upwards eight inches, fresh and healthy appearing, closed with silkworm-gut sutures, opening into the peritoneal cavity; the coils of gut immediately adjacent were covered with bloody fibrin. The subject was very stout, and the adipose tissue over the thorax and abdomen was one and one-half inches thick. The contents of the thorax were not remarkable, except that the endocardium was strongly blood-stained. The spleen was small, and rather soft. The kidneys were large, somewhat soft and pale, and contained small cavities, making the texture spongy. The liver was of ordinary size and showed evident fatty infiltration. Numerous areas of fat-necrosis occurred in the abdominal adipose tissue, in the mesentery down to the mesorectum, in the omentum, many about the left kidney; but they were most numerous and extensive about the pancreas. There was no appreciable fluid in the lesser peritoneal cavity. The pancreas was enlarged, firm, dark, red to brownish-black, mottled; hemorrhage was evident; there were numerous thrombi in the medium-sized and small veins but not in the main arteries or veins. The duct of Wirsung was rather small, but was patulous, joining with the common bile-duct just before entering the duodenum. A probe was passed through the orifice of the diverticulum of Vater with moderate force. There were no calculi in the diverticulum or gall-ducts, and the latter were patulous; the gall-bladder contained several calculi from one-eighth inch in diameter to the size of a cherry; it was adherent to the colon. Attempts were made to dissect out the solar plexus, with indifferent success; however, it was observed that hemorrhage proceeding from the pancreas had extended to the vicinity of the solar plexus. The brain and spinal cord were not examined. Cultures from the general peritoneal cavity yielded a small motile bacillus, probably b. proteus; cultures from the lesser peritoneum gave no growth; cultures from the gall-bladder yielded a streptococcus. The pancreas itself contained b. coli communis in pure culture. Sections of the pancreas showed numerous bacilli of similar morphology and some very large bacilli. To a considerable extent these bacilli were growing within small cavities that seemed to be lymph-spaces and sometimes small veins. The bacilli were most numerous in the head of the pancreas. The bacilli seen here were probably partly b. coli communis and partly a gas-forming organism, the latter being responsible for the cavities seen in the kidney, mentioned above. The veins of the kidney and often the walls of these cavities showed numerous large Gram-positive bacilli. It is not unlikely that they were b. aerogenes capsulatus, but as no anaërobic cultures were made a decisive opinion cannot be given. In sections of the pancreas its histology was hardly recognisable in many places, owing to the absence of nuclear stain; in occasional fields the structure was quite well preserved. In all the ducts observed the epithelial lining was wanting, and they contained desquamated epithelium and debris. Other prominent features were firm thrombi in the veins, hemorrhage, fat-necrosis and the presence of great numbers of dark pigment granules. In the head of the pancreas and in the fibrous tissue of the duodenum adjacent to it, numerous phagocytic cells enclosing yellowish pigment masses occurred; they were interpreted as indicating that some hemorrhages had occurred at a time prior to the final acute attack. The epithelial lining of the duct of Wirsung and of the bile-duct was deficient. A small duct, passing through the wall of the duodenum, supposed to be the duct of Santorini, contained desquamated epithelium in quite good condition. With the exception of an excessive degree of fatty infiltration of the liver, and the large bacilli above mentioned in connection with the kidney, the histology of the other organs was not important. The necrosis of the pancreas and hemorrhage were the most striking features of this case. By some this would probably be called gangrenous pancreatitis.

Case VI.—For the clinical history of this case we are indebted to Dr. James P. Barr. J. B., male, age 35, a shoemaker, whose health had previously been good, was seized with pain in the abdomen on June 28, 1907, 10 A. M. The pain was located over the stomach, a little to the left of the median line, extending to the left side and back. At 12 M. his temperature was 100°; pulse, 90; at 9 P. M., temperature, 101°; pulse, 120. There was vomiting. During the day morphine was given on account of the severity of the pain. June 29, 7 A. M., temperature, 102°; pulse, 150; he was then in great pain; at 12 M., temperature, 102.5°; pulse, 160; he had slight convulsive attacks. At 10. the pulse was very weak and rapid; he was in great pain, and was perspiring freely; he had convulsions and was at intervals delirious. A probable diagnosis of intestinal obstruction was made, and he was conveyed to the Buffalo General Hospital, where his temperature was found to be 106°, pulse, 150; blood examination, only leukocyte count made, 9,600. The abdomen was tympanitic but not rigid; examination of heart and lungs showed nothing abnormal; he was unconscious; the condition appeared like shock; operation was considered hopeless. At 3 A. M., June 30, his temperature was 109.8°; pulse, 185. His death occurred shortly afterwards, about forty-one hours after the first symptom was said to have been experienced.

Autopsy, June 30, 3:30 P. M., about twelve hours after death. The body was that of a short, well-built man, showing the usual rigor mortis and post-mortem lividity; there was slight jaundice of the skin and sclerotic of the eye. The adipose tissue beneath the skin and in the omentum and mesentery was well developed but not excessively. The brain was not examined. The organs of the thorax showed no condition of importance. The spleen and the kidneys were somewhat small and pale, but not otherwise remarkable. There was no exudate on the surface of the peritoneum; the cavity contained a few ounces of turbid fluid; the coils of intestine were distended but not reddened. There was no fat-necrosis to be seen from the general peritoneal cavity.

The lining of the stomach was swollen, hyperemic, and showed some small hemorrhages. The lining of the duodenum was swollen and hyperemic; the lining of the upper part of the jejunum was swollen; the remainder of the intestine with the vermiform appendix, was normal. There were no ulcers; there were no calculi in the intestines; the contents were yellowish-white and semifluid. The liver was large, soft, yellow, a little mottled with red, and appeared fatty and somewhat jaundiced. The gall-bladder was moderately distended with green bile; there were no calculi in the gall-bladder or ducts; the ducts were patulous. On moderate pressure on the gall-bladder bile issued from the bile-papilla, which was prominent. The duodenum was ligated and filled with eosin solution; on pressure the fluid could not be made to regurgitate into the diverticulum of Vater.

On opening the lesser peritoneal cavity it was seen that the posterior wall of the cavity, covering the anterior surface of the pancreas and the adjacent portion of the stomach, had a curious white, opaque appearance, as though acted on by some corrosive. The cavity contained one to two ounces of turbid fluid, with some white flakes. On section it was seen that the adipose tissue in and about the pancreas showed extensive fat-necrosis, not in the form of nodules, but spreading diffusely or in little streaks. This condition was very marked behind the head of the pancreas, where the tissues appeared to be infiltrated with soft purulent material, extending to the root of the mesentery. Microscopic examination showed the material to contain drops of fat and many needle-shaped crystals, and a few bacilli, resembling b. coli communis, but no leukocytes. The pancreas was of normal size, grayish pink in color, quite firm towards the tail, softer near the head. A small hemorrhagic area was seen behind the middle of the pancreas. The duct of Wirsung opened into the diverticulum of Vater about one-fourth inch from its orifice; its interior was not bile-stained, but was entirely filled with opaque, soft, white material, which appeared under the microscope to be amorphous, with an occasional leukocyte, and a considerable number of bacilli resembling b. coli communis. The duct was rather small in caliber. The peculiar white character of its contents appeared to be shared by the wall of the duct, and to extend a short distance into the surrounding tissue. The papilla for the duct of Santorini was found in its usual situation. In sections, a channel supposed to be this duct was subsequently found; it was not obstructed and its epithelium was in good condition. No thrombosis or other obstruction of the bloodvessels of the pancreas was noted.

Cultures were made from the intestine, gall-bladder, spleen, and the fluid in the lesser peritoneal cavity. From the first three was obtained a moderate sized short bacillus, with rounded ends, little or slightly motile, Gram-negative, producing gas in dextrose and reducing neutral red, and producing gas and acid in lactose; it was regarded as being b. coli communis or b. lactis aerogenes and was not studied further. The lesser peritoneum yielded a nonmotile bacillus, usually short oval, sometimes longer, often in pairs, which did not produce acid or gas in glucose or lactose, did not reduce neutral red. It was doubtfully and slightly Gram positive. The presence of bacilli having the morphology of b. coli communis in the duct of Wirsung and the fat-necrosis about the head of the pancreas has been noted.

In sections of the pancreas no bacteria could be found, but the examination was rendered quite uncertain by the presence of many granules apparently precipitated by the fixing fluids (formaldehyde and especially by alcohol). Sections of the pancreas at various points showed an imperfect nuclear stain or often none at all in most of the tissue, when only the outlines of the acini and lobules remained distinguishable. Fat-necrosis was very extensive between the lobules and on the surface. The connective tissue of the surface at various points showed distinct increase in the number of its cells, marked accumulations of leukocytes; small hemorrhages were also present and some fibrin. That the changes in the pancreas did not occur post-mortem, chiefly at least, was evident from the changes just noted. The wall of the duct of Wirsung gave no nuclear stain; the epithelium was deficient; the duct was completely filled with amorphous material taking the eosin stain; rarely leukocytes occurred in the contents.

Histological examination of the heart, lungs, and spleen showed nothing of importance. In the kidney the epithelium of the convoluted tubes stained poorly. As to the liver, the cells were slightly fatty; some of them showed granules of yellow pigment, and some of the fine bile-capillaries contained yellow pigment masses: the bile ducts were normal; the connective tissue was moderately infiltrated with leukocytes. The stomach showed marked hyperemia and some small hemorrhages in the mucosa; the epithelial cells were granular, many were desquamated; many showed karyokinetic figures. The duodenum showed an increased number of leukocytes in the fibrous layers; there was slight desquamation of the epithelium of Brunner's glands. The impression obtained from the condition of the pancreas in this case was that necrosis was the most conspicuous feature, while hemorrhage and inflammatory phenomena were comparatively unimportant.

The possibility that the process in the pancreas was due to infection and was secondary to gastro-duodenitis has considerable evidence in its support. Nothing was found at the autopsy to account for the very high temperature of the patient's last hours. It is to be regretted that the brain could not be examined.

Case VII.—For the history of this case, we are indebted to Dr. Roswell Park. "M. S., age 47. The patient was sent to the Buffalo General Hospital by Dr. Buswell with a statement that he had long suffered from gallstone disease and would require operation. He was having attacks of peritoneal and paroxysmal pain along with nausea and vomiting. Altogether his sickness covered a period of nine years. Two or three years ago Dr. Buswell urged operation which had been postponed, until now apparently it was forced upon him when he was not in good shape. It was after another acute attack of pain that I operated.

"November 21, 1907, I explored the upper abdomen in the expectation of finding most serious trouble in or about the gall-bladder. On opening the peritoneum I was confronted by a mass of adhesions binding together all the upper viscera, obscuring details and making it extremely difficult to decide even what would be advisable. I could not find the gall-bladder. While down in the depths I felt a hardened mass which suggested cancer of the pancreas. While separating adhesions here, there came a gush of pus and necrotic tissue, having that in it which was much like both fat and fecal matter, and it was evident that I had gotten into some old abscess whose minute origin was unknown. It seemed wise to stop at this point. Accordingly, I drained the abscess cavity and had to lightly pack the wound because of the tendency to oozing.

"Patient steadily ran down and died on the third day after the operation. It seemed to me in the uncertain past there had been ulcer of the duodenum, with perforation, and the abscess cavity into which I had opened was the consequence of old adhesions and perforation, while the material it contained was of uncertain age, but old. The mass was large enough to press upon the biliary passages and to cause all sorts of disturbances."

Autopsy, November 25, 1907, about six hours after death, by Dr. N. G. Russell. The subject was jaundiced, adipose tissue moderately developed. The thoracic organs were not remarkable. The surface of the abdomen showed a wound about seven inches long to the right of the rectus muscle, from which a dark, foul smelling fluid issued. On opening the peritoneal cavity, the adipose tissue of the omentum and mesentery presented numerous opaque grayish spots from the size of a pin-head to a bean which proved to be areas of fat-necrosis. Similar nodules occurred about the left kidney in the adipose tissue, which was abundant. Fat-necrosis was not present in the region opened at the operation. The upper part of the peritoneum had numerous adhesions and some turbid fluid. The spleen was not remarkable. The kidneys were large, pale, narrow cortex, capsule stripping easily; bladder normal. The lower part of the intestines was not remarkable.

The stomach contained mucus and reddish fluid. The stomach was glued to the diaphragm by moderately fresh adhesions; rupture of these adhesions opened a pocket of bloody pus. The duodenum showed a round ulcer five-eighths inch in diameter, two and one-half inches below the pylorus, and a second round ulcer one-half inch in diameter, three and three-fourths inches below the pylorus. The lower of these ulcers was situated just to the left of the papilla for the duct of Santorini. This ulcer had perforated the wall of the duodenum and communicated with the lesser peritoneum.

The surface of the head of the pancreas and the adjacent portion of the body were marked by discoloration, red, gray to black in color. The neighboring adipose tissue showed opaque, white or gray areas of fat-necrosis. The lesser peritoneum contained turbid fluid. The substance of the pancreas appeared firm, pink and normal, except superficially in the region of discoloration and necrosis above mentioned. No thrombi were seen in the large vessels. The bile-papilla was prominent. The mouth of the diverticulum of Vater was open and large enough to admit a lead pencil. The common bile duct was open and enlarged to the same size; its walls were thick. The duct of Wirsung opened just within the mouth of the diverticulum and readily admitted a match. A probe was easily passed through the duct nearly to the tail of the pancreas. The lining of the duct was not bile-stained. Neither the diverticulum of Vater nor the ducts contained calculi. The gall-bladder was imbedded in a mass of adhesions, extending between the liver and omentum, forming a dark mass from which foul smelling fluid issued. The external surface of the gall-bladder appeared necrotic; its walls were thick; it contained soft granular material, in all making a mass the size of the finger. The liver weighed four and one-half pounds, and was yellowish in color. The gastro-hepatic omentum contained a pocket of pus, of the size of a hen's egg. No cultures were secured. Sections made subsequently showed numerous bacilli of various sizes on the upper surface of the pancreas. The same surface was covered with granular material, fibrin, and leukocytes.

The capillaries were often dilated and filled with blood. The adjacent part of the pancreas contained an unusual amount of fibrous tissue containing numerous mononuclear cells of various sizes, (partly plasma-cells and mast-cells), also polynuclear leukocytes. The acini of this portion of the pancreas had the appearance of being compressed and atrophied. Small groups of acini, located superficially, failed to take the nuclear stain, and were in some cases nearly disorganized. A single good-sized vein was found which was completely thrombosed. The impression derived from the histological examination, was that the surface of the pancreas next to the lesser peritoneum had been affected for sometime by a chronic inflammatory process to which a severe, acute, infectious inflammatory process had recently been added. The duct of Santorini contained in a few places a small number of polynuclear leukocytes; its epithelium, and the epithelium of its branches, was in good condition. There was no evidence that the inflammatory process had spread to the pancreas through this duct. The deeper portion of the pancreas was normal; the epithelium of the ducts was in good condition; no bacteria were found within or without the ducts. No reasonable doubt can exist that the superficial pancreatitis present in this case was the result of the perforation of the duodenal ulcer immediately over it. The pancreatitis was certainly not the sole cause of death, and was probably not the principal cause.

*General Remarks.*—The earlier histories of Cases IV. and V. are at least strongly suggestive that the final attack of pancreatitis was not the first attack of pancreatitis from which the patient had suffered; the histology of the cases gives some support to this suggestion. In subjects dying from other causes we have several times seen evidence that some degree of pancreatitis had existed previously.

In a recent paper, W. J. Mayo[2], in speaking of acute and sub-acute pancreatitis says: "There is undoubtedly an exaggerated idea as to the fatality of fat necrosis." It also appears from the clinical histories that cases may go through their entire course with little indication of infection. Egdahl[3] concludes, from experiments performed by him recently, that the collapse symptoms of acute pancreatitis are due to poisons derived from broken down pancreatic tissue, which is similar to the view held by Doberauer. Guleke[4] believes that these symptoms are due to absorption of the secretion of the pancreas. Sailer[5] was unable to demonstrate toxic properties in emulsions of necrosed pancreatitis tissue injected into guinea-pigs. Sailer also produced pancreatitis in dogs by the injection of oil into the duct of Wirsung, with ligation of the duct; the blood of such dogs proved to be toxic for guinea-pigs.

The severity of the symptoms may seem to be out of all proportion to the extent of the lesion, as in Case VI., although it must be admitted that the evidence in this case lacks completeness in the absence of an examination of the brain. In our previous series we reported a case (CASE I.[6]) in which evidence of inflammation and hemorrhage of the pancreas were lacking, but which we now regard as having been necrosis of the pancreas. Inflammatory exudation, hemorrhage, and necrosis of the pancreas seem to be combined in varying proportions in acute pancreatitis.

---

2. Journal American Medical Association, April 11, 1908.
3. Egdahl. Journal Experimental Medicine, Vol. IX., p. 385.
4. Archiv f. klinische Chirurgie, Vol. LXXXV. p. 615, 1908.
5. Transactions Association American Physicians, 1908.
6. American Journal Medical Sciences, 1895, Vol. CX., p. 251.
Transactions Association American Physicians, 1895, Vol. X.

The necrosis of the whole wall of the duct of Wirsung in Case VI. and the presence of soft, amorphous white substance in it are noteworthy.

*Remarks on Etiology.*—It now seems certain that the cause of acute pancreatitis is not the same in all cases, and the fact is illustrated by the four reported above. We propose to discuss the most important of the causes that have been suggested, and their bearing upon our cases:

1. Traumatism has been shown to be the cause of acute pancreatitis in a number of instances. Case VII. may conveniently be placed in this group, as the duodenal ulcer practically operated as a mechanical injury, though in combination with infection. Case III. of our previous series we believed to be due to unavoidable manipulation during an operation on the bile-passages.

2. Gallstones. The association of gallstones with pancreatitis has been noted by many writers, although some have denied that any relationship exists. Three of the four cases in this series had gallstones. Egdahl[7] and Williams and Busch[8] found gallstones recorded as present in about 40 per cent. of cases of pancreatitis. The mechanism described by Opie[9] is well known. We could find no evidence that this mechanism operated in any of our four cases. Johnstone[10] has recently described four cases of hemorrhagic pancreatitis in which the duct of Wirsung and the bile-duct opened separately, so that any mechanical relation, with gallstones was obviously impossible. Williams and Busch[11] have recently proposed another mechanism to account for the association of pancreatitis with gallstones. They suggest that the passage of a gallstone may so dilate the orifice of the diverticulum of Vater, that intestinal contents may be injected into the duct of Wirsung. This suggestion is not incompatible with the view of Hlava (supported by Flexner's experiments), that pancreatitis may be caused by the injection into the duct of Wirsung of gastric juice.

In our Case VII., the orifice of the diverticulum of Vater was dilated and wide open. The suggestion of Williams and Busch derives a certain amount of support from this case, as it shows that the anatomical conditions required for their mechanism may, in practice, exist; however, the thin wall of the pancreatic duct would probably operate as a valve and prevent injection of intest-

---

7. Bulletin Johns Hopkins Hospital, Vol. XVIII., 1907.
8. Journal Medical Research, Vol. XVII., 1907.
9. A small gallstone blockiug the orifice of the diverticulum of Vater with injection of bile into the duct of Wirsung. Opie. Diseases of Pancreas, J. B. Lippincott & Co., 1903, p. 118,
10. University of Colorado Medical Bulletin, February, 1907.
11. Journal of Medical Research, Vol. XVII., 1907.

inal contents into this duct in many cases. As a matter of fact, although the anatomical conditions in Case VII. seemed perfect for the injection of intestinal contents into the duct of Wirsung, we are convinced that in this case, the pancreatitis was due to the perforation of a duodenal ulcer.

3. Infection is given high rank as a cause of acute pancreatitis by some writers, as will be seen by reference to the recent work of Robson and Cammidge[12].

In our Case VI. there was considerable evidence in favor of the supposition that the pancreatitis arose from infection from the duodenum by way of the duct of Wirsung. In Case VII. which we attribute to the perforation of a duodenal ulcer, infection doubtless played a part, bacteria having access to the surface of the pancreas through the ulcer.

In Cases IV. and V., as in our previous series, although bacteria were present in the pancreas, we are uncertain how much influence they exercised upon the process.

In this connection it may be of interest to refer to the claim of some writers that pancreatitis may be associated with mumps.

4. Gastroduodenal catarrh, with or without the abuse of alcohol, is often mentioned as a cause of pancreatitis. It probably operates indirectly. It was a prominent feature in our Case VI. Egdahl[13] suggests that intestinal inflammation may close the orifice of the diverticulum of Vater and lead to retrojection of bile.

5. Causes inherent in the pancreas itself.—The sudden onset and rapid course of acute pancreatitis, without adequate explanation in many or most cases, have impelled investigators to look for a cause in some perverted action on the part of the ferments of the pancreas. This class of causes is discussed at length in the paper of Williams and Busch already referred to. The outlook for a solution of the problem along these lines seems the more promising when two other factors are considered. (A). The necrosis of pancreatic tissue usually seen in acute pancreatitis, closely resembles the post-mortem autodigestion of the pancreas seen in ordinary subjects. (B). The fat-necrosis which so commonly accompanies acute pancreatitis has been demonstrated within all reasonable probability to be caused by escape of the fat-splitting ferment of the pancreas.

Causes of this class are not likely to be discovered through pathological anatomy, but will probably need to be worked out by animal experiments.

6. Arteriosclerosis, embolism, and thrombosis of bloodvessels have been suggested as causes; they have not seemed important

12. The Pancreas, its Surgery and Pathology. 1907.
13. Bulletin Johns Hopkins Hospital, Vol. XVIII., 1907.

factors in our experience. Such thrombosis of bloodvessels as we have seen in our cases, we have regarded as secondary result of the affection of the pancreas. Levin concluded from his recent work on experimental injuries of the pancreas that the effects on the organism were gravest, where the most serious interference with the circulation of the organ was combined with injury[14].

14. Journal Medical Research, Vol. XVI., 1907.

